# APP Knockout Mice Experience Acute Mortality as the Result of Ischemia

**DOI:** 10.1371/journal.pone.0042665

**Published:** 2012-08-09

**Authors:** Maya A. Koike, Alexander J. Lin, Jonathan Pham, Elaine Nguyen, James J. Yeh, Rombod Rahimian, Bruce J. Tromberg, Bernard Choi, Kim N. Green, Frank M. LaFerla

**Affiliations:** 1 Institute for Memory Impairments and Neurological Disorders, University of California Irvine, Irvine, California, United States of America; 2 Laser Microbeam and Medical Program, Beckman Laser Institute and Medical Clinic, Irvine, California, United States of America; 3 Department of Biomedical Engineering, University of California Irvine, Irvine, California, United States of America; 4 Edwards Lifesciences Center for Advanced Cardiovascular Technology, University of California Irvine, Irvine, California, United States of America; Hertie Institute for Clinical Brain Research, University of Tuebingen, Germany

## Abstract

The incidence of Alzheimer’s disease increases in people who have had an ischemic episode. Furthermore, APP expression is increased following ischemic or hypoxic conditions, as is the production of the Aβ peptide. To address the question of why APP and Aβ are increased in hypoxic and ischemic conditions we induced an ischemic episode in APP knockout mice (APP−/−) and BACE1 knockout mice (BACE−/−). We find that both APP−/− and BACE−/− mice have a dramatically increased risk of mortality as a result of cerebral ischemia. Furthermore, APP knockout mice have reduced cerebral blood flow in response to hypoxia, while wild-type mice maintain or increase cerebral blood flow to the same conditions. The transcription factor, serum response factor (SRF), and calcium-binding molecule, calsequestrin, both involved in vascular regulation, are significantly altered in the brains of APP−/− mice compared to wild type controls. These results show that APP regulates cerebral blood flow in response to hypoxia, and that it, and its cleavage fragments, are crucial for rapid adaptation to ischemic conditions.

## Introduction

Alzheimer’s disease is a progressive neurodegenerative disease that affects the elderly. The disease is thought to occur due to the accumulation of the Aβ peptide in the aging brain. The accumulation of Aβ in soluble oligomers and extracellular plaques initiates a cascade of downstream events, culminating in synaptic and neuronal toxicity [1]. This loss of synapses and neurons likely causes the memory loss and cognitive decline associated with the disease [2]. Aβ is produced by sequential proteolytic cleavage of its parent holoprotein, the amyloid precursor protein (APP) by β-secretase activity followed by γ-secretase cleavage. The majority of disease modifying therapeutics are aimed squarely at inhibiting the cleavage of APP by its respective proteases, however, little is known about the endogenous function of APP and what consequences modulating its cleavage and expression will have in the aged brain.

APP is produced in most of the tissues in the body, is evolutionarily conserved and has two mammalian APP-like family members, APLP1 and APLP2, suggesting that APP plays important biological roles. There is evidence that APP and its cleavage fragments are involved in a number of crucial cellular functions including cell survival, neurite outgrowth, synaptogenesis, synaptic plasticity, memory, neurogenesis, cell adhesion, and neuroprotection [3]–[6]. Notably, mice in which APP or BACE1 expression has been reduced or knocked out have only subtle phenotypic alterations including reduced size, reduced grip strength and locomotor activity [7], [8]. Most likely, the lack of overt deficits is due to compensation by APLP1 and APLP2, as knocking out APP, APLP1 and APLP2 or APP and APLP2 results in postnatal lethality, although it has been reported that there is not an upregulation in APLP’s in the APP knockout mice [9]–[11]. Additionally, the lack of observable impairment in the APP−/− only mice may also be due to APP being necessary in response to cellular stresses, thus not being critical under normal conditions. Interestingly, APP and Aβ are upregulated following a number of cellular stressors including ischemia, hypoxia, excitotoxin injection, traumatic brain injury and heat shock (reviewed in [3] [12]).

To determine whether APP−/− and/or its cleavage products are an integral part of a metabolic stress response, we subjected APP−/− mice to a bilateral common carotid artery occlusion resulting in global ischemia. Strikingly, whereas the control mice survived 48-hours following a global ischemic injury, both the APP−/− and BACE−/− mice experienced a mortality rate of around 60%. Additionally, we found that the APP−/− mice show a dysregulated vascular response to hypoxia and altered proteins associated with vascular changes. Together, our data indicate that APP and/or its cleavage fragments play a necessary role in response to cellular stressors like global ischemic injury.

## Materials and Methods

### Mice

All mouse experiments were performed in accordance with animal protocols approved by the Institutional Animal Care and Use Committee, University of California, Irvine. The animal approval number is 2011-2974. APP−/−, BACE−/− and their control non-transgenic mice C57BL/6J were obtained from Jackson Laboratories (Bar Harbor, Maine) strain names B6.129S7-App^tm1Dbo^/J, B6.129-Bace1^tm1Pcw^/J. All three strains of mice are on the C57BL/6J background.

**Figure 1:**
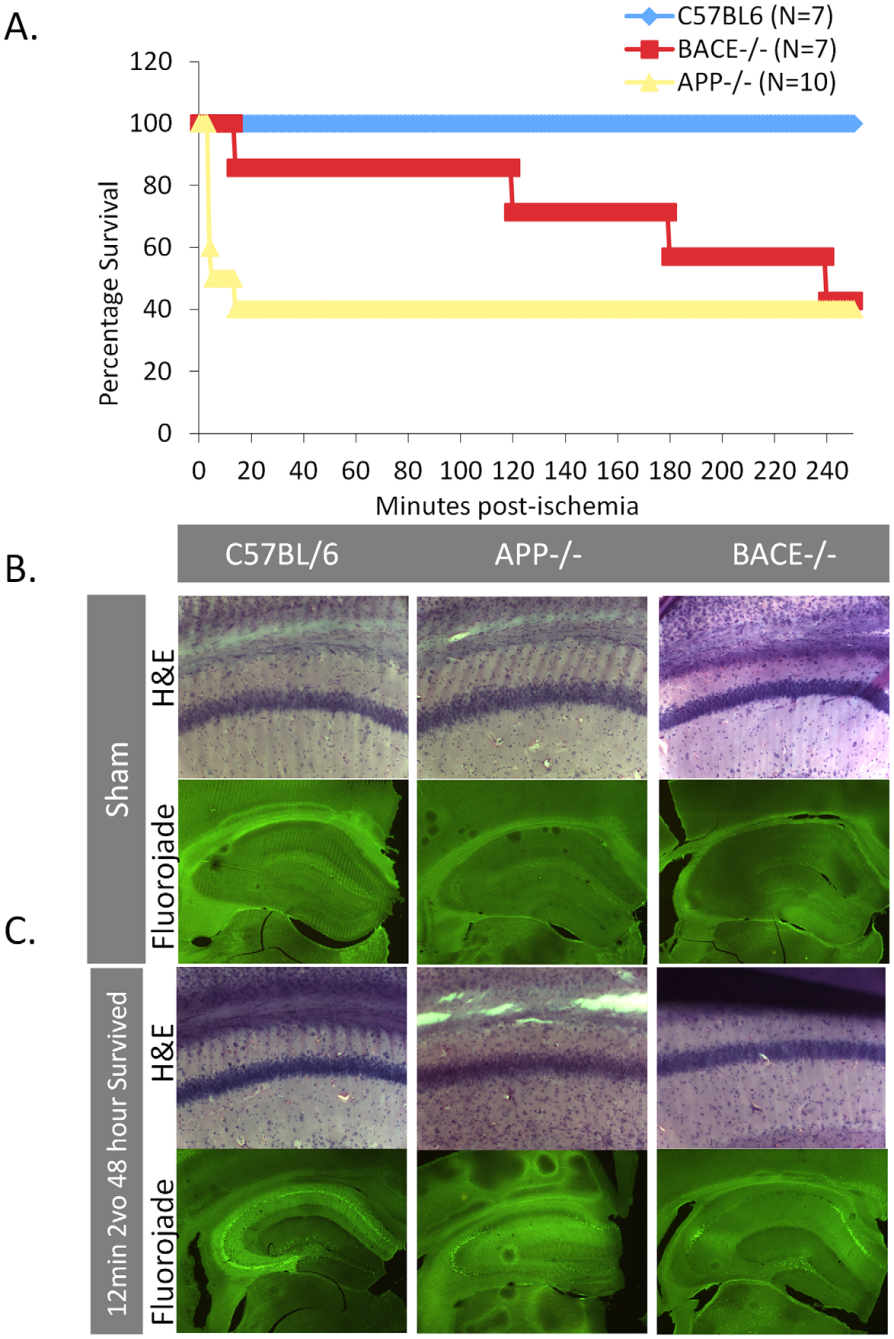
Ischemia induces increased mortality in APP−/− and BACE−/− mice. Mice were subjected to a 12-minute bilateral common carotid artery occlusion. (A) None of the control mice (blue line), but 60% of the APP−/− mice (yellow line) and 60% of the BACE−/− mice (red line) died during the 12-minute ischemic insult or within 4-hours of the injury. (B, C) Fluoro-Jade and H&E staining in hippocampal regions of control, APP−/− and BACE−/− subjected to a sham surgery or a 12-minute common carotid artery occlusion and survived 48-hours later. There was no increase in cell death within the hippocampus between the APP−/−, BACE−/− mice and the non-transgenic controls that had been subjected to sham surgeries or the mice that survived the 12-minute surgery (2vo) and were sacrificed 48-hours after injury.

**Figure 2:**
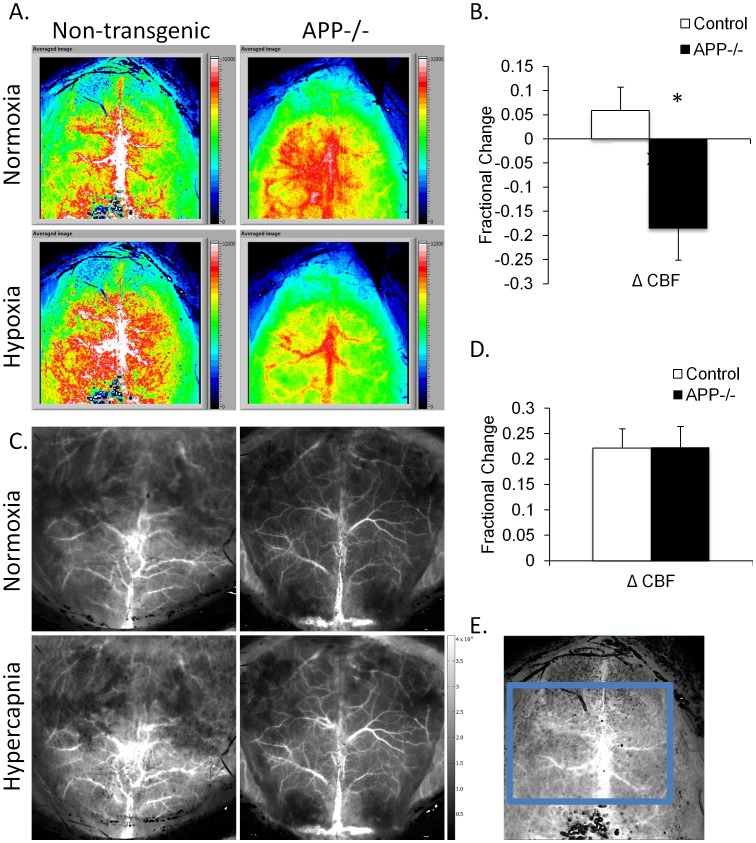
Hypoxia alters blood flow in APP−/− mice. (A) Blood flow as detected by laser speckle imaging (LSI) decreased in the APP−/− mice but increased in the non-transgenic control mice subjected to a hypoxic challenge. (B) Quantification of the average change in blood flow measured by LSI revealed a significant difference between the reaction of the APP−/− mice and the control mice to hypoxia. (C) There was no difference in the reaction of the APP−/− mice and control mice to hypercapnia measured by LSI, fractional change was confirmed by quantification (D). (E) Demonstration of the area of interest quantified in each animal.

### Imaging the Circle of Willis

To perform a gross check of the vasculature of the Circle of Willis we injected carbon black in a modified protocol [13], [14]. Each mouse was fully anesthetized and the sternum was opened revealing the heart. The mouse was first perfused with phosphate buffered saline followed by perfusion with 1–2 ml of 5% carbon black via the left ventricle (with a cut made in the right atrium to allow blood release). The brain was then carefully removed and imaged using a dissecting microscope.

### Global Cerebral Ischemia

Global cerebral ischemia was induced by transient, bilateral clamping of the common carotid arteries for 12-minutes (two vessel occlusion -2VO) as previously described [15], [16]. If the animals died during or within 48-hours of the surgery the brains were collected as soon as possible. Surviving mice were sacrificed using CO_2_ and their brains were collected. An additional group of no-injury control APP−/− and non-transgenic mice were sacrificed and brain, heart, muscle and blood samples were collected.

**Table 1:** Hemoglobin chromophore response to hypoxia.

Inhaled gas	Mice	ctO_2_Hb (µM)	ctHHB (µM)	TotHb (µM)	O_2_ Sat (%)
21% O_2_	Ctrl	113±10	40±3	153±12	74±0.8
21% O_2_	APP−/−	105±12	35±3	140±15	75±0.4
5% O_2_	Ctrl	80±10	73±3	153±12	52±3
5% O_2_	APP−/−	72±15	67±8	139±21	50±4

**Table 2:** Hemoglobin chromophore response to hypercapnia.

Inhaled gas	Mice	ctO_2_Hb (µM)	ctHHB (µM)	TotHb (µM)	O_2_ Sat (%)
21% O_2_	Ctrl	104±7	32±0.7	136±7	77±1.2
21% O_2_	APP−/−	100±4	39±5	139±4	72±3
5% O_2_	Ctrl	114±5	29±0.2	143±5	80±0.7
5% O_2_	APP−/−	111±4	32±3	143±5	78±2

### Phenytoin Injection

25 APP−/− mice were injected intraperitoneally with phenytoin or Saline as a control (Sigma Aldrich, St. Luis MO) at 30 mg/kg IP in 1∶1 PBS and DMSO, 1-hour before global ischemia surgery (n = 13 saline injected and n = 12 phenytoin injected).

### Hypoxia Challenge

Nontransgenic (n = 4) and APP−/− (n = 4) mice with exposed skulls were placed in an imaging setup while breathing normal air (21% oxygen, balance nitrogen) under 1% isoflurane anesthesia from a gas mask. Baseline images were taken for 3-minutes before switching the inhaled gas mixture to hypoxic air (5% oxygen, balance nitrogen) for 5-minutes.

### Hypercapnia Challenge

Using the same setup as for the hypoxia challenge, a second group of 4 C57BL/6J and 4 APP−/− mice were imaged at baseline for 5-minutes followed by hypercapnic gas (5% carbon dioxide, balance air) for 7-minutes.

Animals were sacrificed using pentobarbital overdose and their brains were collected. All brain samples were cut in half sagittally. Half of the samples were preserved in paraformaldehyde and the other half flash frozen.

**Figure 3:**
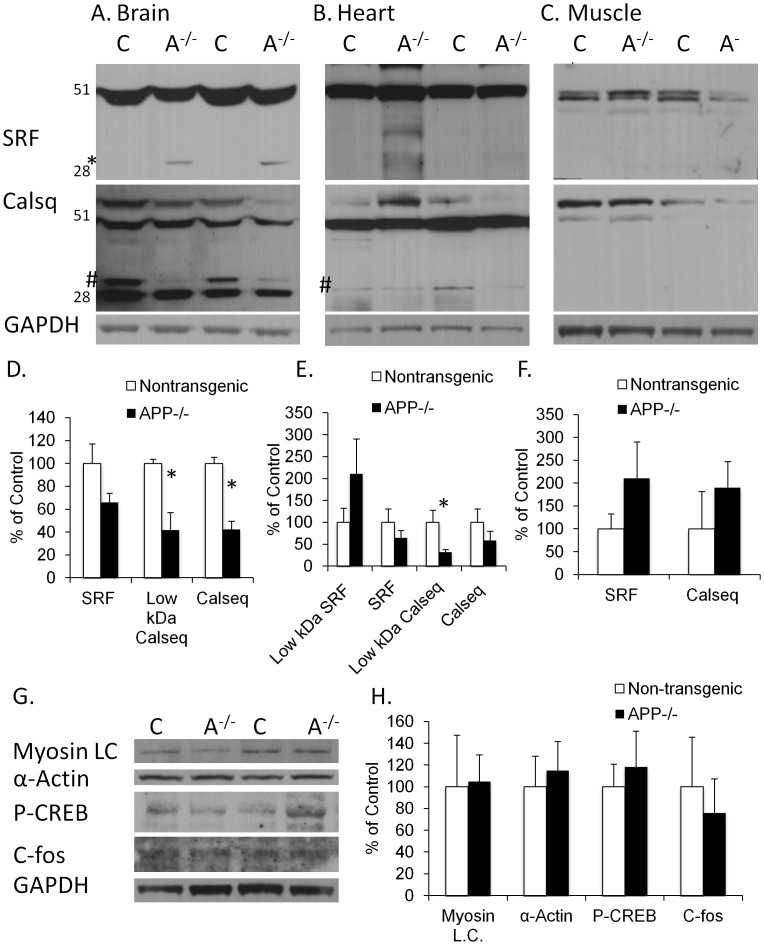
SRF and calsequestrin levels are altered in APP−/− mice. (A) Western blot analysis of mouse brain tissue (Control  =  C, APP−/−  =  A−/−) reveals a significant increase in a 32 kDa molecular weight band of SRF in the APP−/− (please see * besides the SRF). There is a significant decrease in the levels of full-length and a low molecular weight band (please see #) of calsequestrin in the brains of the APP−/− mice (A). (D) Quantification of SRF and Calsequestrin in the brain homogenate normalized to GAPDH and represented as a percentage of Control. (B) There was also a significant decrease in the low molecular weight band (please see #) of calsequestrin in the hearts of the APP−/− mice. (E) Quantification of SRF and Calsequestrin in the heart homogenate normalized to GAPDH and represented as a percentage of Control. (C, F) We did not find any significant differences between in the muscles of APP−/− and control mice in either protein. (G) Western blot analysis revealed no significant changes in other SRF targets. All western blots were normalized to GAPDH and represented as a percentage of control.

### Imaging

Laser speckle imaging (LSI) and spatial frequency domain imaging (SFDI) were used to study blood-flow dynamics and hemoglobin oxygenation states, respectively, associated with the respiratory challenges. These methods have been previously described in detail [17], [18]. Cranial imaging windows over the mouse forebrain were created by surgically removing skin over the region of interest (bregma to lambda, bilaterally to the temporalis muscle attachments). Immediately after exposing the region of interest the mouse’s head was secured by ear bars to prevent motion and heavy mineral oil was thinly spread on the mouse’s skull to keep it from drying. Raw speckle images were collected during baseline and after hypoxia/hypercapnia. Images were converted to Speckle Flow Index (SFI) maps using a previously-described algorithm [19]. Concurrently, SFDI images were acquired at 670 nm and 850 nm and converted to oxy- (ctO_2_Hb) and deoxy- (ctHHb) hemoglobin concentration maps on a pixel-by-pixel basis [20]. Total hemoglobin (TotHb) was calculated as the sum of ctO_2_Hb and ctHHb, and tissue oxygen saturation (O_2_ Sat) was calculated as 100*ctO_2_Hb/TotHb. The mean flow rate and hemoglobin concentrations were calculated from a region of interest selected over the mouse forebrain as described above.

### Immunohistochemistry and Confocal Microscopy

Light-level immunohistochemistry was performed with an avitin–vitinerimmunoperoxidase technique (ABC kit; Vector Laboratories Inc, Burlingame, CA) and visualized with diaminobenzidine as previously described [15]. Fluoro-Jade staining, a selective stain for degenerating neurons, was performed as previously described [21] (N = 7 control mice and 10 APP−/− mice).

Fluorescent immunolabeling was performed using free-floating sections as previously described [15]. The prevent signal bleed-through, all fluorophores were excited and scanned separately using lambda strobing.

### Western Blotting

Western Blotting (N = 4 control mice and 4 APP−/− mice) was performed as described previously [22]. Protein extracts were prepared from complete half brain samples cut sagittally, homogenized in T-per (Pierce Biotechnology, Rockford, IL) extraction buffer and Complete Mini Protease Inhibitor Tablets (Roche, Indianapolis, IN) followed by high-speed centrifugation at 100,000× g for 1 hour. The supernatant was taken as the soluble protein extract. Protein concentrations were determined by the Bradford method. Equal concentrations of protein were separated by SDS-polyacrylamide gel electrophoresis on a 4 to 12%, 12%, or 10% Bis/Tris gel using 2-(N-morpholino)ethanesulfonic acid (MES) or 3-(N-morpholino)propanesulfonic acid (MOPS) running buffer (Invitrogen, Carlsbad, CA). They were then transferred on to 0.2 µM^2^ nitrocellulose membranes, blocked for 1 hour in 5% (v/v) nonfat milk in Tris-buffered saline (pH 7.5) supplemented with 0.2% Tween20. The blots were then probed for the primary antibody of interest overnight, washed 3 times in Tris-buffered saline (pH 7.5) supplemented with 0.2% Tween20 then exposed the the appropriate secondary antibody for one hour at room temperature. The membrane was then washed 3 more times and then developed using Pico Chemiluminescent Substrate (Pierce, Rockford, IL) and imaged on a Mini Medical Film Processor (AFP Imaging Corp, Elmford, NY). Quantitative densiometric analyses were performed on digitized images of immunoblots with ImageJ. All error bars represent SEM.

### Statistical Analyses

All data were analyzed by Student’s two-tailed t-tests. Results were considered significant only when p<0.05. Outliers greater than 3x the standard deviation of the mean were removed from statistical analysis. All Western blots were normalized to GAPDH. Error bars indicate standard error of the mean (SEM).

## Results

### Mice Deficient in Either APP or BACE1 Experience an Increase in Acute Mortality as the Result of Global Cerebral Ischemia without an Increase in Cell Death

A number of studies have found elevated levels of AD-related proteins such as APP and Aβ acutely following cerebral hypoperfusion or hypoxic injuries [23], [24]. To determine whether APP or its cleavage fragments were crucial during ischemic injury, we induced a global cerebral ischemic insult in APP−/−, BACE1−/− mice and control non-transgenic mice. Notably, none of the non-transgenic mice died during or within 48-hours of the surgery, but the APP−/− and the BACE1−/− mice lost about 60% of their cohort during this time period (Fig. 1A). Of note, the majority of the APP−/− mice died during the surgery whereas the BACE−/− mice died after reperfusion.

We examined the brains of the mice to determine if the mortality we observed in the APP−/− and BACE−/− mice resulted from a gross increase in cell loss following ischemia. We found no clear differences in cell morphology, measured by H&E stain, or cell death, measured by Fluoro-Jade staining, between the control and knockout mice in the cohort that survived the surgery and were sacrificed 48-hours after injury (Fig. 1B–C). These findings demonstrate that the acute mortality experienced by the knockout mice is likely not due to global cerebral-cell death.

### Phenytoin does not Alter Ischemia-induced Mortality

Previous studies reported that BACE−/− mice are more susceptible to seizures and more susceptible to cell loss and acute mortality during pharmacologically-induced seizures [25], [26]. We initially hypothesized that ischemia led to seizure-like activity in the transgenic mice and this resulted in the observed mortality. However, when we injected the antiepileptic drug, Phenytoin (30 mg/kg), intraperitoneally one hour prior to the ischemic surgery in a separate cohort of APP−/− mice (n = 13 saline injected and 12 phenytoin injected), we did not find that this drug paradigm had an effect on ischemia-related mortality. Seven of the saline injected and 6 of the phenytoin injected mice died, respectively (data not shown).

### APP−/− Mice have Dysregulated Cerebral Blood Flow during Hypoxia, but Vascular Tone is Preserved in Hypercapnia

To determine if APP−/− mice had an altered response to vascular insults we exposed APP−/− and C57BL/6J mice to either hypoxic or hypercapnic conditions while imaging the brain (Fig. 2E). Spatial frequency domain imaging (SFDI) data showed a 22 and 25% reduction in tissue oxygen saturation levels in control and APP−/− mice, respectively, during hypoxic challenge (Table 1). Whereas the control mice experienced a 5% increase in blood flow on hypoxic exposure, the APP−/− mice had an 18% decrease in blood flow as measured by laser speckle imaging (LSI) (Fig. 2A–B). Notably, when vasodilation was tested with ample oxyhemoglobin present during hypercapnia challenge (Table 2), we did not find a significant difference between the blood flow response of the APP−/− mice and the C57BL/6J mice (Fig. 2C–D). These results indicate that the altered vascular response exhibited by the APP−/− mice may be connected to the lack of oxygen rather than a ubiquitous attenuated vascular response.

### APP−/− Mice have Altered Serum Response Factor and Calsequestrin

Based on the data that APP−/− mice have altered vascular response to low oxygen environments, we explored the protein levels of Serum Response Factor (SRF), a transcription factor involved in vessel contraction, which is upregulated in APP overexpressing mice and in AD patients [27]. While close, we found no significant decrease (p = 0.06) in full-length SRF in the brains of APP−/− mice compared with the C57BL/6J mice (Fig. 3A, D). Notably, we found a significant increase in the levels of a 32 kDa band of SRF in the brain of the APP−/− mice not present in the C57BL/6J controls, which has been previously described as a dominant negative form of SRF and shown to contribute to heart failure (Fig. 3A, p = 0.01) [28]. We also looked at the levels of calsequestrin, one target of SRF also involved in vascular response [27] and found a reduction in steady state levels in APP−/− brains (Fig. 3A, D). Upon longer exposure, we also found a decrease in a low molecular weight band of calsequestrin in the brain of the APP−/− mice compared with controls (Fig. 3A, D see #). As APP is present in a range of tissue types we sought to determine whether knocking out APP produced similar effects in other highly vascularized tissues. Notably, we found that the same low molecular weight band of calsequestrin was reduced in the heart of APP−/− mice although we did not observe significant changes in full-length calsequestrin (Fig. 3B,E # indicates low molecular weight band). We did not find any significant differences in SRF or calsequestrin in the muscles of the APP−/− and control mice indicating that this may be a tissue specific alteration (Fig. 3C, F).

We next assessed other SRF target genes in the brains of the APP−/− and control mice. We did not find any significant differences between levels of any target proteins including the vascular-related Myosin light chain and α-actin or the immediate early genes phosphorylated CREB and C-fos (Fig. 3 G, H).

## Discussion

APP, and Aβ production, has been widely reported to be upregulated following a number of metabolic insults and stresses [3]. In particular, hypoxia and ischemia have been linked to increases in APP expression and Aβ production [23], [24]. Based on these observations we asked the question of why APP and Aβ would be upregulated in ischemic/hypoxic conditions, and hypothesized that APP might be a stress response protein that is beneficial in a hypoxic/ischemic environment. To test this hypothesis we gave bilateral common carotid occlusions to wild type mice, and mice lacking either the APP or BACE1 genes. Notably, we found that the APP−/− mice and BACE−/− mice suffered from acute mortality during and after the occlusion period, which was not seen in the wild type control animals. This result suggests that APP or its cleavage fragments is beneficial in an ischemic environment, and that its absence can be fatal. Curiously, most mortality in the APP−/− cohort occurred during the occlusion period, rather than afterwards, when reperfusion injuries are likely to occur. It is unclear how APP affects the ischemic environment, or how the lack of APP leads to acute mortality, but these observations may have implications in AD patients treated with Aβ and APP targeted therapies as strokes and ischemic episodes are frequent in the elderly population. While we have employed a global ischemic model in our experiments it is important to note that patients can also undergo focal ischemic insults, such as focal embolic and thrombolytic strokes [29], and the middle cerebral artery occlusion (MCAO) model would better recapitulate this scenario [30], [31]. However, global ischemic insults are also common in the elderly and arise from conditions such as hypotensive injuries [31], spontaneous subarachnoid hemorrhage [32], anesthesia related incidents [33] and cardiac arrest [31], [34], thus our experiments utilizing global ischemia model these conditions [35]. Future experiments will determine how APP processing affects outcome following focal ischemic events, while our current results clearly show a role for APP in the response to global ischemia.

We found that acute mortality was not directly related to tissue damage, as Fluorojade staining was equivalent between APP−/−, BACE−/− and wild type mice subjected to the bilateral occlusions. Given that all of the mice are on the same C57BL/6J background, it is unlikely that our results are due to vascular differences between the mice. However, it is possible that is that the lack of APP or BACE1 induces structural changes in the brain during development, which we are unaware of, and that these changes render the animals susceptible to ischemia induced mortality. We cannot discount this possibility, but until conditional APP−/− mice are generated we are unable to rule this out. Of note, and with a similar observation to our study, two groups have published data showing that BACE1−/− mice were more susceptible to spontaneous and pharmacologically-induced seizures as well as acute mortality as the result of induced seizures in a subset of the animals [25], [26]. Interestingly, we also observed spontaneous seizure-like activity in the APP−/− mice, especially during the stress of a cage change (data not shown). However, injecting the anti-epileptic drug phenytoin did not alter mortality outcome.

Using Laser Speckle Imaging and Spatial Frequency Domain Imaging, we examined vascular responses in the APP−/− and control mice during a hypoxic and hypercapnic insult. We found that during hypercapnia challenges the dilatory and contractile mechanisms were intact. However, performing hypoxia challenges revealed a decrease in blood flow in the APP−/− mice but not the C57BL/6J mice. This altered vascular response suggests that APP plays a role in the vascular response to hypoxic stress. Additionally, we found alterations in some proteins involved in vessel contraction; however additional experiments are necessary to determine the role of protein alterations in these mice.

Our study indicates that APP or an APP cleavage fragment is an integral part of stress response. However, more research is necessary to determine which fragment is involved and what the molecular pathways are underlying the relationship between the lack of APP and/or its fragments and mortality during ischemic stress. Understanding this interaction is of clinical value given the interest in both APP fragments as drug targets for AD, and given the high rate of cerebral hypoperfusion injuries in the elderly population.
